# A Temperature-Compensated Single-Crystal Silicon-on-Insulator (SOI) MEMS Oscillator with a CMOS Amplifier Chip

**DOI:** 10.3390/mi9110559

**Published:** 2018-10-29

**Authors:** Mohammad S. Islam, Ran Wei, Jaesung Lee, Yong Xie, Soumyajit Mandal, Philip X.-L. Feng

**Affiliations:** Department of Electrical Engineering & Computer Science, Case School of Engineering, Case Western Reserve University, Cleveland, OH 44106, USA; msi16@case.edu (M.S.I.); rxw210@case.edu (R.W.); jxl803@case.edu (J.L.); yxx510@case.edu (Y.X.)

**Keywords:** oscillator, resonator, micro/nanoelectromechanical systems (MEMS/NEMS), application-specific integrated circuit (ASIC), MEMS-ASIC integration, programmable sustaining amplifier, single-crystal silicon (SC-Si), silicon-on-insulator (SOI), real-time temperature compensation loop

## Abstract

Self-sustained feedback oscillators referenced to MEMS/NEMS resonators have the potential for a wide range of applications in timing and sensing systems. In this paper, we describe a real-time temperature compensation approach to improving the long-term stability of such MEMS-referenced oscillators. This approach is implemented on a ~26.8 kHz self-sustained MEMS oscillator that integrates the fundamental in-plane mode resonance of a single-crystal silicon-on-insulator (SOI) resonator with a programmable and reconfigurable single-chip CMOS sustaining amplifier. Temperature compensation using a linear equation fit and look-up table (LUT) is used to obtain the near-zero closed-loop temperature coefficient of frequency (TC*f*) at around room temperature (~25 °C). When subject to small temperature fluctuations in an indoor environment, the temperature-compensated oscillator shows a >2-fold improvement in Allan deviation over the uncompensated counterpart on relatively long time scales (averaging time τ > 10,000 s), as well as overall enhanced stability throughout the averaging time range from τ = 1 to 20,000 s. The proposed temperature compensation algorithm has low computational complexity and memory requirement, making it suitable for implementation on energy-constrained platforms such as Internet of Things (IoT) sensor nodes.

## 1. Introduction

Stable oscillators are vital for precision timekeeping in various applications, including wired and wireless communications, positioning, navigation, and sensing. In particular, oscillator stability against temperature variations and fluctuations is critical for many of these applications [1,2]. Temperature-compensated quartz crystal oscillators provide excellent stability versus temperature, and have thus dominated the timing and frequency control market for decades. However, they are not suitable for monolithic integration with CMOS circuitry, which makes them sub-optimal for emerging applications such as mobile devices and IoT nodes, where miniaturization is important for reducing cost. Thus, there has been a strong push towards miniaturized alternatives. Oscillators referenced to micromachined resonators [2,3,4,5] are promising alternatives due to their small form factors, low phase noise thanks to high *Q*, low power consumption, good long-term stability, compatibility with batch processing, high reliability, low cost, and wide operating temperature range. It is important to select the right fabrication material in order to optimize such resonators for a particular application. For example, silicon carbide (SiC) has outstanding mechanical or thermal properties, which makes SiC devices suitable for high temperature applications. Here we focus on silicon (Si) devices, since they can be easily integrated with CMOS technology, making them attractive for low-cost applications. Moreover, earlier work has demonstrated the excellent short-term stability of oscillators based on Si MEMS resonators [5]. However, the elastic properties of Si are strongly temperature-dependent, so temperature fluctuations degrade the long-term frequency stability of Si-based oscillators. A number of compensation methods have been reported for improving temperature stability [2,6,7,8,9,10,11,12,13]. For example, in [12], the authors implement a passive compensation method by utilizing silicon dioxide (SiO_2_), which has an opposite TC*f* compared to the structural material of the main resonator (Si); this compensation technique results in a parabolic (second-order) frequency dependence versus temperature. More recently, researchers have proposed the use of multiple temperature compensated resonators with different TC*f* values to generate a temperature-stable frequency output [13]. These studies, however, have been focused on passive temperature compensation, which increases the complexity of the MEMS design, and therefore, the overall cost of the system.

**Figure 1:**
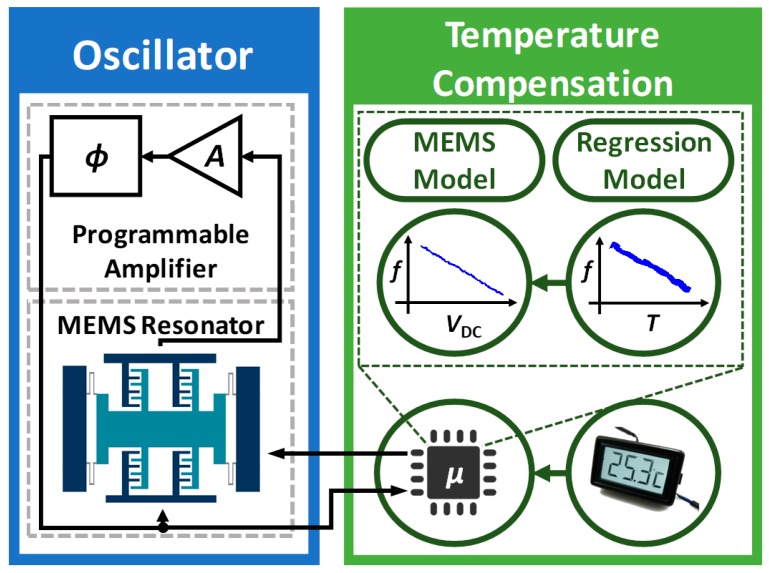
Overview of the temperature-compensated oscillator.

In this paper, we describe an active real-time software-controlled approach for temperature compensation of MEMS-referenced oscillators (see Figure 1). In particular, we demonstrate our approach using a high-*Q* single-crystal (SC) Si comb-drive MEMS resonator interfaced with a programmable single-chip CMOS sustaining amplifier. The uncompensated oscillator has a negative TC*f*, i.e., its oscillation frequency decreases as the temperature increases. In addition, the resonant frequency increases if the DC polarization voltage decreases. We have built a linear regression model based on training data to capture these phenomena, and then used them to develop a real-time temperature compensation loop. The loop is based on (i) simultaneously sampling the oscillator frequency and ambient temperature using a frequency counter and a microcontroller unit (MCU); and (ii) changing the DC polarization voltage to cancel the expected frequency variation. This straightforward technique significantly improves the long-term stability of the oscillator, thus making it suitable for many emerging fundamental and industrial applications [14,15,16,17,18,19,20].

## 2. System Architecture of the SOI MEMS Resonator and CMOS Sustaining Amplifier

### 2.1. Overview of the SOI MEMS Resonator

**Figure 2:**
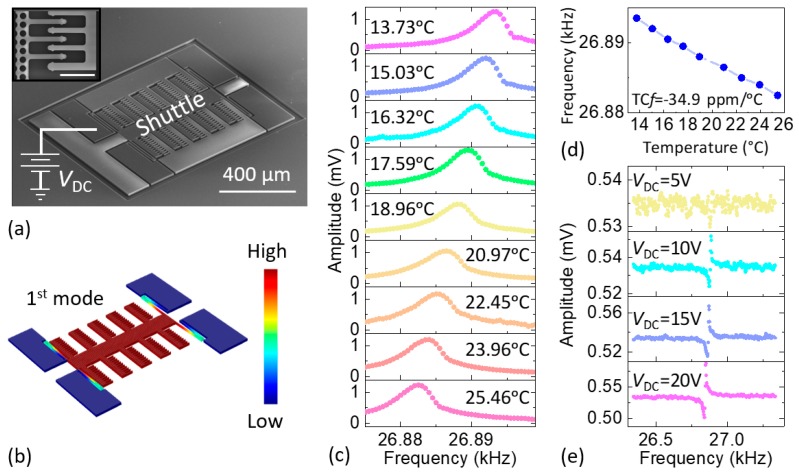
Single-crystal Si-on-insulator (SOI) comb-drive MEMS resonator characteristics. (**a**) Scanning electron microscopy (SEM) image. The inset shows a partial zoom-in view of the comb drive and fingers. Scale bar: 20 μm; (**b**) Simulated vibration pattern and mode shape for the first in-plane resonance mode; (**c**) Optically measured transmission data and frequency response around the first resonance as temperature increases; (**d**) Open-loop resonance frequency dependence on temperature for the extraction of TC*f* from the data in (**c**); (**e**) Electrically measured transmission and frequency response when various values of the DC polarization voltage are applied to the shuttle. The responses in (**c**,**e**) have not been converted to dimensionless transfer functions since the measurement path includes both electrical and optical components, which makes it difficult to derive absolute calibration factors.

We use SC-Si MEMS comb-drive resonators manufactured in a silicon-on-insulator (SOI) process at wafer scale to ensure high-*Q* and low phase noise. The SC-Si comb drive devices were fabricated on a 4-inch SOI wafer with a top device layer of 12 µm-thick SC-Si and an oxide (SiO_2_) with thickness of 2 µm. We first performed standard photolithography with a resolution of 1 µm to pattern the wafer. The top SC-Si layer not protected by photoresist was etched away by the Bosch process in a deep reactive ion etching (DRIE). The wafer was then diced into 5 mm × 5 mm chips. After etching in buffered oxide etch (BOE) to remove the SiO_2_ under the comb drive shuttle, chips were released in a critical point dryer (CPD) to prevent stiction to the substrate. Finally, aluminum (Al) wire bonding was performed on the selective pads for electrical interfacing. As shown in Figure 2a, the fabricated comb-drive SOI MEMS device has an overall length, width, and thickness of 900 µm, 700 µm, and 20 µm, respectively. In addition, the comb-drive fingers are 2 µm apart. We use finite element method (FEM) simulations in COMSOL Multiphysics to study the vibration pattern and mode shape of the first in-plane flexural resonant mode (see Figure 2b). Figure 2 also summarizes the results from optical characterization of the device resonance, which were obtained by using a custom-built laser interferometer specially engineered for ultrasensitive detection of in-plane resonance modes [21,22]. The optically measured resonant frequency for a DC polarization voltage of *V*_DC_ = 20 V is *f* ≈ 26.8 kHz at room temperature (the measurement system is shown in Figure S1 in the Supplementary Materials), with a quality factor of *Q* = 13,000 at room temperature and in a moderate vacuum of ~5 mTorr. Figure 2c shows the optothermally excited, optically detected resonance responses [21,22] of the device as a function of temperature (at *V*_DC_ = 20 V); we control the latter with a Peltier cooler. We then extract the resonance frequency versus temperature from the optically measured data (see Figure 2d). The figure shows that this MEMS device has a negative TC*f* of −34.9 ppm/°C, which is similar to other SC-Si devices in the literature. Figure 2e shows that the resonance frequency also shifts with the DC polarization voltage. We use these empirical measurement results for temperature compensation, as we shall describe in later sections.

### 2.2. Overview of the Programmable Single-Chip CMOS Sustaining Amplifier

**Figure 3:**
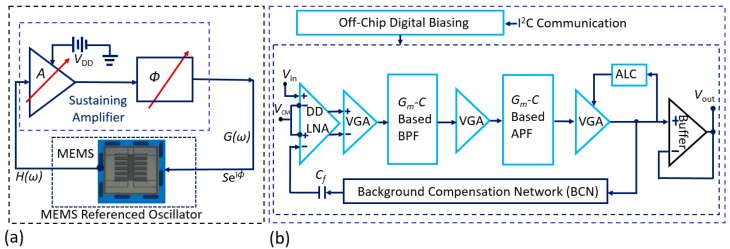
(**a**) Simplified system diagram illustrating the integration of the MEMS resonator chip with the CMOS sustaining amplifier chip; (**b**) Simplified block diagram of the programmable single-chip CMOS sustaining amplifier (corresponding to the same color-coded dashed-line box in (**a**)).

We have designed and fabricated a single-chip sustaining amplifier using the OnSemi 0.5 μm CMOS process and interfaced it with the MEMS resonator as illustrated in Figure 3a. The chip (see Figure 3b) contains a differential-difference low-noise amplifier (DD-LNA), two second-order band-pass filters (BPFs), three all-pass filters (APFs) used as phase shifters, variable gain amplifiers (VGAs), an automatic level control (ALC), a “background compensation network” (BCN) to cancel the parasitic electrical capacitance of the resonator, and an op-amp-based output buffer [23]. Various parameters of these blocks can be set by using programmable current sources implemented on the test board.

To integrate the SOI MEMS chip and the CMOS amplifier chip, we connect the two differential inputs of the DD-LNA to the MEMS resonator and the BCN, respectively. The DD structure eliminates unwanted capacitive coupling between these terminals. The measured 1/*f* corner frequency of the LNA is <10 kHz. We use a gate-input wide-linear-range operational transconductance amplifier (WLR-OTA) as the basic building block for the rest of the circuit. The WLR-OTA uses source degeneration and bump linearization to improve the input-referred linear range (*V*_L_) [24]. We use this OTA to implement second-order *G_m_-C* BPFs that determine the frequency response of the amplifier, i.e., select a particular resonant mode. Each BPF uses two OTAs in a negative feedback loop to gyrate (invert) the impedance of a capacitor to realize an active inductor, since passive on-chip ones are impractical at such low frequencies. A third OTA acts as a variable resistor to form a parallel RLC circuit, and the OTA bias currents are adjusted to set the resulting center frequency and *Q*.

Similarly, we use the OTA to implement *G_m_-C* APFs that control the overall phase shift of the amplifier, which enables the phase criterion for oscillations to be satisfied for the chosen mode. Each APF provides unity voltage gain and a frequency-dependent phase shift of −2tan^−1^ (*ωt*), where *t* = *C*/*G_m_*. Thus, each APF provides a phase shift of 0–180° as *t* is adjusted from 0 to ∞ via an off-chip bias currents. However, in practice, *t* can only be varied over a finite range, which reduces the useful control range to ~120°. Hence three cascaded APFs are used for ~360° control. The maximum signal amplitude for total harmonic distortion (THD) <5% is ≈ 180 mV; this is largely limited by the OTAs.

Four cascaded VGAs control the voltage gain of the amplifier, i.e., enable the gain criterion for oscillations to be satisfied for the chosen mode. Each VGA uses two OTAs to set the gain, and a third OTA to create a high-pass filter. The latter allows the VGAs to be AC-coupled, which prevents accumulation of DC offset and low-frequency interference. The chip also includes an ALC that regulates the oscillator’s output voltage amplitude (*V*_OUT_) by adjusting the VGA gain. This reduces amplitude-to-phase noise conversion and prevents accidental damage to the MEMS resonator due to overload.

The amplitude- and phase-tunable BCN is designed to cancel the parasitic electrical feedthrough of the MEMS resonator. Such feedthrough, which is generally broadband, makes it difficult for the system to oscillate at the true optimal mechanical resonance frequency [25]. This is undesirable, since off-resonance operation degrades close-in (low offset frequency) phase noise. The BCN drives an on-chip capacitor (*C_f_* ≈ 28 fF) that feeds back a compensation signal to the negative input terminal of the LNA.

Electrical characterization of the sustaining amplifier shows that the center frequency and *Q* of the BPF are adjustable from ~10 to ~90 kHz and from 0.5 to 9, respectively, while the APF phase shift can be adjusted over the full 360° range as expected. Moreover, the peak amplifier gain can be adjusted from 0 to 80 dB by using the VGAs, which is sufficient to overcome the transmission loss of typical comb-drive MEMS resonators within this frequency range.

**Figure 4:**
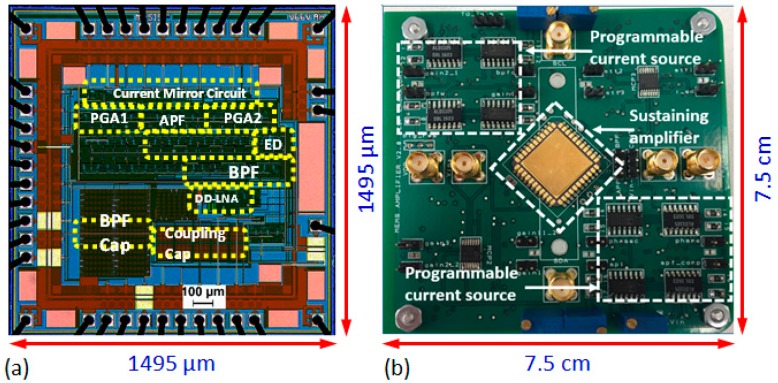
(**a**) Die micrograph of the CMOS sustaining amplifier; (**b**) Test board used for characterizing the amplifier and integrating with the MEMS resonator to build the oscillator.

The entire amplifier core occupies a layout area of 1150 μm × 1150 μm, and operates at a supply voltage of 3.3 V. Figure 4a shows a die micrograph of the sustaining amplifier. The input-referred noise of the amplifier with the BPF center frequency set to *f* = 26 kHz is ~7.2 nV/Hz^1/2^, which agrees with circuit simulations and corresponds to an equivalent noise resistance of *R_n_* = 4 kΩ at room temperature. Moreover, *R_n_* is typically much smaller than *R_m_*, the motional resistance of comb-drive MEMS resonators in this frequency range. Thus, the close-in phase noise of the oscillator will be dominated by the high-*Q* resonator, as desired. Details of the measured specifications and performance of the chip are summarized in Table 1.

## 3. Oscillator Referred to the Single-Crystal SOI MEMS Resonator

### 3.1. Oscillator System

**Figure 5:**
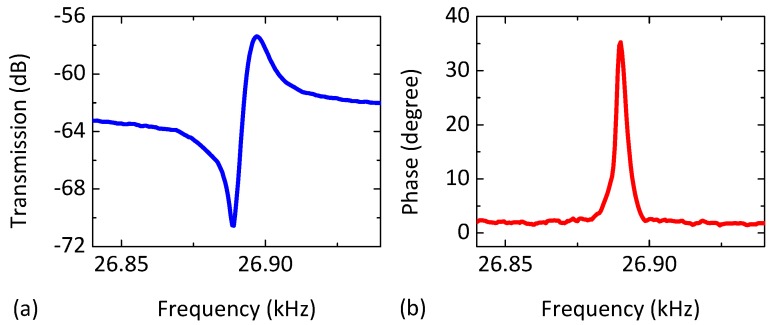
Electrical characterization results of the single-crystal SOI MEMS resonator. (**a**) Measured transmission (dB) and frequency response of the resonance; (**b**) Open-loop phase (degrees) around the first mode for *V*_DC_ = 20 V and an input power of −10 dBm.

The packaged sustaining amplifier chip is mounted on a test board designed to fit inside a vacuum chamber (~5 mTorr, room temperature) along with the SOI MEMS resonator. The board only needs four connections: power, output, and a two-wire I^2^C bus. An external MCU uses the bus to program 16 on-board digital potentiometers. The latter are combined with op-amps to realize programmable bias current generators that, in turn, set all on-chip parameters with 8-bit precision. Figure 5 shows the measured open-loop feed-forward transmission (magnitude and phase) using the MEMS and sustaining amplifier for a DC polarization voltage of *V*_DC_ = 20 V and a drive amplitude of −10 dBm. We then create a MEMS-referenced oscillator by programming the BPFs, APFs, and VGAs to realize enough gain and the correct phase near resonance. In addition, we program the ALC to set the output amplitude to *V*_OUT_ ≈ 400 mV.

**Figure 6:**
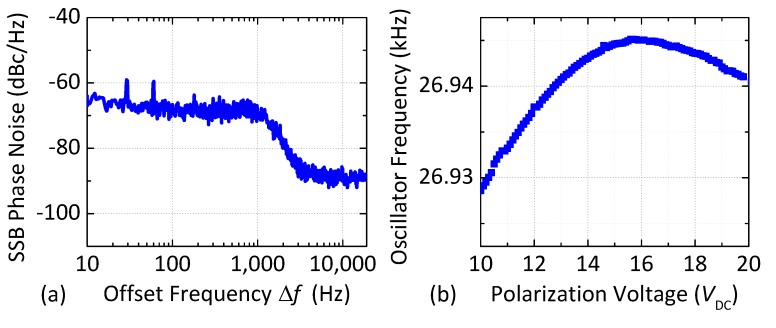
(**a**) Measured single-sideband (SSB) phase noise of the single-crystal SOI MEMS oscillator for *V*_DC_ = 20 V; (**b**) Measured electrical tuning of the oscillator frequency as a function of *V*_DC_ (approximately −1 Hz/V for voltages >16 V).

The performance of the oscillator is monitored by using a spectrum analyzer (Agilent 4395A, Keysight Technologies, Santa Rosa, CA, USA) and a frequency counter (Agilent 53132A, Keysight Technologies, USA). The measured single-sideband (SSB) phase noise spectrum of the MEMS-referenced oscillator at ambient temperature is shown in Figure 6a, while the dependence of the oscillation frequency on the DC polarization voltage *V*_DC_ is shown in Figure 6b. Note that the oscillation frequency is a non-monotonic function of *V*_DC_.

### 3.2. Temperature Compensation of the MEMS-Referenced Oscillator

**Figure 7:**
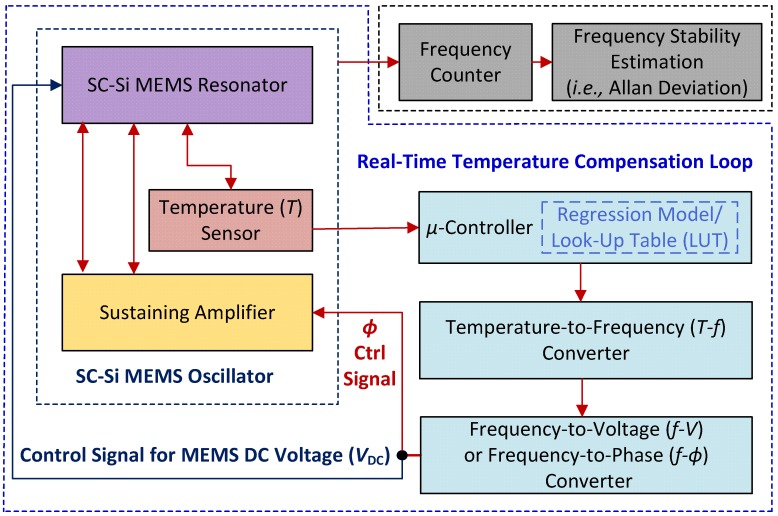
Block diagram of the model calibration procedure, and the proposed real-time temperature compensation loop.

Figure 7 shows the block diagram of the overall real-time temperature compensation loop (including calibration of the oscillator using a linear model). The overall real-time temperature compensation loop (i.e., data acquisition, calibration, post-processing, and command execution) has been implemented in a software environment (MATLAB, R2018a). The oscillator is placed inside a vacuum chamber as shown in the left side of the block diagram along with a digital temperature sensor (MCP9808, Microchip Technology, Chandler, AZ, USA) with a resolution of ±0.25 °C. The sensor is directly attached to the DIP24 package containing the wire-bonded resonator to ensure that it measures device temperature. A miniaturized development board (Arduino Uno R3, Arduino, New York, NY, USA) based on an ATmega328 MCU (Atmel Corporation, San Jose, CA, USA) with 32 KB memory acts as the system controller. The MCU is used to program 16 on-board digital potentiometers using a 2-wire I^2^C bus and also acquires data from the temperature sensor. A MATLAB script stores all real-time experimental *T-f* data vectors and sends commands to set the desired MEMS *V*_DC_ voltage with a resolution of 5 mV using a source meter unit (Keithley 2450, Agilent Technologies, Santa Clara, CA, USA) programmed over GPIB. In addition, the MATLAB script implements temperature-to-frequency (*T-f*) and either frequency-to-voltage (*f-V*) or frequency-to-phase (*f-ϕ*) conversion functions to realize the proposed temperature control loop.

**Figure 8:**
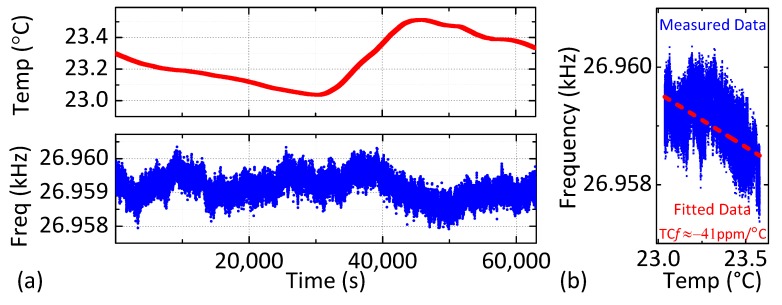
(**a**) Measured temperature variation (top) and fluctuation of oscillation frequency (bottom) for the uncompensated oscillator over 24 h; (**b**) Linear fit of the oscillation frequency versus temperature.

During calibration, the device temperature is accurately set by using a Peltier module (CP08-63-06, Laird Technology, Cleveland, OH, USA) which is powered from an external benchtop DC power supply. The frequency counter has been used to measure the oscillation frequency over a period of seven (7) days with a gate time (*t*_g_) of 100 ms. A data-driven linear model is then derived by using offline post-processing. The best-fitting model given is given by

(1) $$f_{osc}=m\times T+c,$$

where *T* is the temperature (in °C), *f_osc_* is the oscillation frequency (in Hz), *m* is the slope, and *c* is the y-intercept. The coefficients of Equation (1) are computed by averaging the best-fitting values from 6 runs (individual values are shown in Table 2). These coefficients are used for temperature compensation. Specifically, Equation (1) is stored in MCU memory and used to calculate the optimal *V*_DC_ in terms of the measured temperature. Figure 8a shows how the frequency of the uncompensated MEMS-referenced oscillator fluctuates due to room temperature variations; the estimated TC*f* = −41 ppm/°C is extracted from the fitting in Figure 8b. The result is in good agreement with the open-loop resonator TC*f* shown in Figure 2d, which suggests that the closed-loop TC*f* is dominated by the resonator and not the sustaining amplifier. A proportional control algorithm is then used to compensate for the measured TC*f*, and the resulting frequency stability is estimated using the Allan deviation, *σ*_A_(*τ*), as a function of averaging time *τ* [26,27].

**Figure 9:**
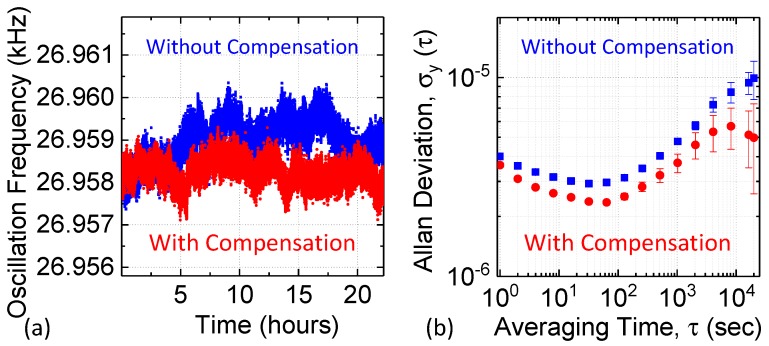
(**a**) Instantaneous frequency of the uncompensated and compensated oscillators over 24 h; (**b**) Measured Allan deviation *σ*_A_ (*τ*) with and without real-time temperature compensation.

Figure 9a,b compare the stability of the temperature-compensated (red curve) and uncompensated (blue curve) oscillators when subject to small indoor temperature fluctuations (approximately ±0.5 °C, detailed statistics are shown in Figure S2 of the Supplementary Materials). The temperature-compensated oscillator achieves a fractional frequency stability of ~3.6 × 10^−6^ in the short-term (*τ* ~1 s), and ~3.7 × 10^−6^ for longer averaging times (*τ* ~1000 s). In the short-term regime (say *τ* < 100 s), temperature compensation noticeably improves the Allan deviation. Long-term drift dominates at averaging time of *τ* > 10,000 s; in this region, Allan deviation is significantly improved by compensating for temperature-related drifts in the resonance frequency. We expect such improvements to persist when the proposed temperature compensation method is extended to large batches of wafer-fabricated resonators, since the resonator TC*f* is mainly determined by the intrinsic material properties of SC-Si.

We have further verified the performance of the proposed compensation method by measuring oscillator frequency stability over a broader temperature range. For this purpose, we have varied the operating temperature of the resonator from 11.2 °C to 41.2 °C by using either a Peltier module (for cooling) or two ceramic space heaters (for heating). The temperature compensation loop is also modified to control both the MEMS DC polarization voltage (*V*_DC_) and the feedback phase shift (*ϕ*) of the sustaining amplifier to minimize the resulting frequency fluctuations. This is because the oscillation frequency is a non-monotonic function of *V*_DC_ (see Figure 6b), so it is unsuitable for compensating large temperature variations and drifts. Thus, here we exploit *ϕ* as another degree of freedom for compensation.

**Figure 10:**
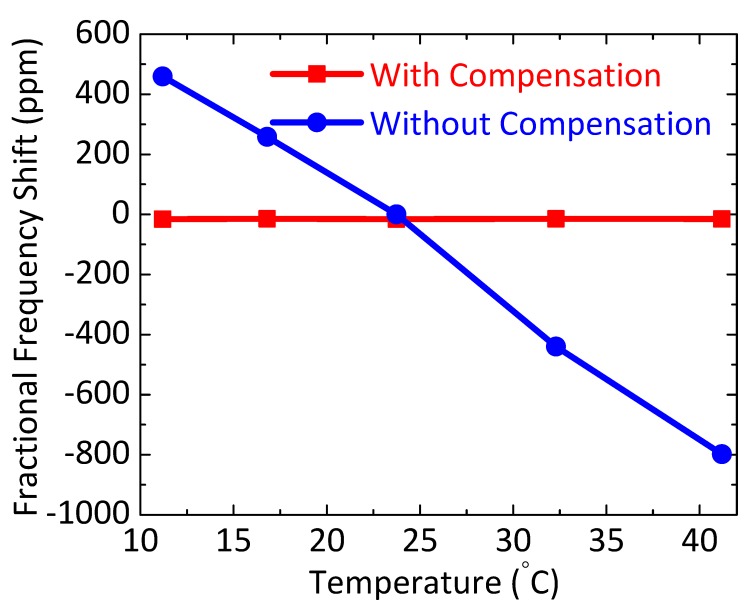
Fractional frequency shift (in ppm) as the device temperature is varied, measured from both the temperature-compensated and uncompensated oscillators over a temperature range of 10 to 45 °C.

Experimental results are shown in Figure 10 (with supporting data shown in Figure Figure S3 in the Supplementary Materials). The results in Table S1 are plotted in Figure 10. The figure shows that the uncompensated oscillator has the expected TC*f* of approximately −40 ppm/°C (dominated by the SC-Si resonator), while the compensated oscillator has near-zero TC*f* (within a measurement error of about ±3 ppm) over this temperature range.

Table 3 compares the performance of the proposed temperature-compensated oscillator with other kHz-range MEMS-referenced oscillators. Our design achieves low close-in phase noise and good long-term stability due to temperature compensation.

## 4. Discussion

Temperature-driven frequency fluctuations are ubiquitous, and are a major performance limiter for SC-Si MEMS-referenced oscillators. Other potential sources of frequency fluctuations include noise sources in the instrumentation [30,31,32], dielectric and charge fluctuations in the resonator, bulk and surface effects in the resonator [33], and limited dynamic range of the sustaining amplifier. The main purpose of the work is to build a temperature-compensated SC-SOI-MEMS oscillator (TCO) for use in low power applications such as IoT sensor nodes. In addition, further improvements in long-term stability can be obtained by replacing temperature compensation with temperature control (i.e., implementing an oven-controlled oscillator (OCO)) and/or by locking the oscillator to an external frequency reference such as GPS over long time scales [34,35]. However, TCOs are more attractive for low-power applications than OCOs because they consume much less power.

## 5. Conclusions

We have demonstrated improved long-term frequency stability by implementing temperature compensation for a single-crystal SOI MEMS-referenced oscillator with a reconfigurable CMOS sustaining amplifier chip. A software-defined compensation loop has been developed for this purpose. Experimental results confirm the effectiveness of the approach in significantly reducing the Allan deviation on time scales *τ* > 10,000 s. These results open up new potential applications for MEMS-referenced oscillators, including high-precision sensing, environmental sensing and monitoring, next-generation wireless communication, and navigation.

## Figures and Tables

**Table 1:** Summary of the electrical specifications and performance of the programmable sustaining amplifier.

Chip Components	Performance
Low-Noise Amplifier (LNA) (for *I*_B_ = 2.5 µA)	Gain: 12 dB; Bandwidth: ~1 MHz Thermal Noise PSD: 13 nV/Hz^1/2^ 1/*f* Corner Frequency: <10 kHz
Band-Pass Filter (BPF)	Center Frequency (*f*_0_): 2–90 kHz; *Q*: 1–8 Dynamic Range (DR): 60.7 dB (24 kHz, *Q* = 2) Linear Range: 500 mV (THD < 5%, 24 kHz, *Q* = 2)
All-Pass Filter (APF)	Phase Control: 0–360° Phase Control Sensitivity: ~0.6°/nA
Variable Gain Amplifier (VGA)	Settable Gain: 0–80 dB
Automatic Level Control (ALC)	Amplitude Control Voltage (*V*_ED_): 0–0.5 V ED Time Constant: 8-Bit control
Background Compensation Path	Gain Control: −20 to 40 dB Phase Control: 0–180°

**Table 2:** Look-up table (LUT) used for temperature compensation.

Day #	Fitted Coefficients	
	*m*	*c*
1	−1.022	27,035
2	−1.169	26,964
3	−1.459	27,067
4	−1.128	26,997
5	−1.1329	27,044
6	−1.1458	26,968
Mean value	−1.1761	27,012

**Table 3:** Comparison of performance between the temperature-compensated oscillator in this work and other MEMS-referenced oscillators in the same frequency range.

Properties	This Work	[28]	[29]	[3]
Resonator Type and Material	Single-Crystal SOI Lateral Comb-Drive	3C-SiC, Comb-Drive	Capacitive Transduction H-Shaped Tuning Fork	Poly-Si Two-Port, Folded-Beam, Comb-Drive
Modes of Oscillation	3	3	1	1
Oscillation Frequencies (*f_osc_*)	~27.0 kHz (Mode 1 Only)	27.1 kHz, 30.3 kHz, 24.2 kHz	32.768 kHz	16.5 kHz
*Q*-Factor	13,000	13,550,10,300 and 9480	52,000	23,400
CMOS Sustaining Amplifier Chip	0.5 μm CMOS	Discrete Components	180 nm CMOS	CMOS Transimpedance Amplifier (TIA)
Die Size	1.5 mm × 1.5 mm	Discrete Components	1.55 mm × 0.85 mm	420 µm × 320 µm (Resonator Only)
Supply Voltage (*V*_DD_)	3.3 V	3.0 V	1.4–4.5 V	2.5 V
SSB Phase Noise	−65 dBc/Hz @ 10 Hz Offset	−78 dBc/Hz @ 12 Hz Offset	Not Reported	−72 dBc/Hz @ 1 kHz Offset (Simulated)
FoM *	133.61	Not Reported	Not Reported	Not Reported
Startup Time	~600 µs	Not Reported	0.2 s	Not Reported
Real-Time Temperature Compensation	Yes	No	Yes	No
Temperature coeficients of frequency (TC*f*)	−34 ppm/°C (MEMS only); <±3 ppm (TCO) over 11.2 °C to +41.2 °C	24.75 ppm /°C ** (rocking vibration mode of MEMS) over 26.85 °C to +426.85 °C	±100 ppm (MEMS only); ±3 ppm (TCXO) & 100 ppm max (XO) over −40 °C to +85 °C	−10 ppm/°C (MEMS only) over 26.85 °C to +96.85 °C
Year	2018	2009	2015	1999

* $FoM=20\log\left(\frac{\omega_{0}}{\Delta \omega }\right)-L(\Delta \omega )-10\log\left(\frac{P_{C}}{1\;m\;W}\right)$, ** ANSYS finite element simulation results.

## Supplementary Materials

The following are available online at http://www.mdpi.com/2072-666X/9/11/559/s1, Figure S1. Schematic of the optical measurement system used for characterizing the open-loop resonance response and TC*f* of the single-crystal SOI MEMS comb-drive resonator. Figure S2. Power spectral density (PSD) and stability estimation: (a) PSD of uncompensated and compensated oscillation frequency; (b) PSD of ambient temperature fluctuations; (c) Allan deviation of uncompensated and compensated oscillation frequency; and (d) Allan deviation of the temperature data shown in (b). Figure S3. Oscillation frequency traces versus time, measured at five different temperature values set and controlled by the sample heating and cooling modules, for oscillators (a) without and (b) with temperature compensation, respectively. Inset plot in (b) shows zoomed-in view of the red box in (b). Table S1. Fractional frequency shift with and without temperature compensation.
