# Supplementary material for: A Temperature-Compensated Single-Crystal Silicon-on-Insulator (SOI) MEMS Oscillator with a CMOS Amplifier Chip
Source: Micromachines (Basel). 2018 Oct 29;9(11):559. doi: 10.3390/mi9110559 (PMC6266509; doi:10.3390/mi9110559)
Supplement: Supplementary file 1 [file micromachines-09-00559-s001.pdf]

micromachines

Supplementary Materials

A Temperature-Compensated Single-Crystal Silicon-on-Insulator (SOI) MEMS Oscillator with a CMOS Amplifier Chip

Mohammad S. Islam, Ran Wei, Jaesung Lee, Yong Xie, Soumyajit Mandal, and Philip X.-L. Feng

S1. Optical Measurement System for Open-Loop Resonance Characterization

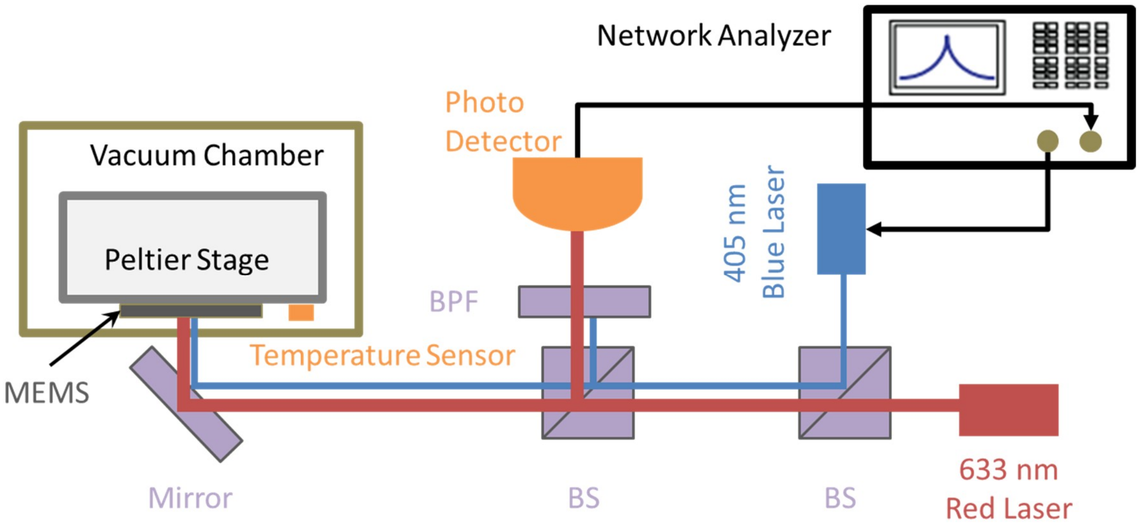

Figure S1. Schematic of the optical measurement system used for characterizing the open-loop resonance response and TC*f* of the single-crystal SOI MEMS comb-drive resonator.

Figure S1 shows the optical interferometry system used for measuring open-loop resonance response and calibration of the TC*f* of the resonator. The modulated 405 nm blue laser optothermally excites the device with sweeping frequency and amplitude that are controlled by the output of the network analyzer. The 633 nm red laser is employed in the ultrasensitive interferometry readout scheme, in order to detect the response, and its output is converted back to an electrical signal by a photo detector and then measured by the network analyzer. BS denotes a beam splitter, while BPF stands for a band-pass filter that allows the red laser to pass through while blocking the blue laser. A Peltier cooler is used to control the temperature of the MEMS device within the vacuum chamber, which is monitored by a sensor installed on the cold surface of the Peltier near the device. The temperature sensor is wired to a digital display outside the vacuum chamber for temperature readout. The device temperature is controlled by adjusting the power supply voltage of the Peltier (outside the vacuum).

S2. Power Spectral Density (PSD) and Stability of Instantaneous Frequency and Temperature

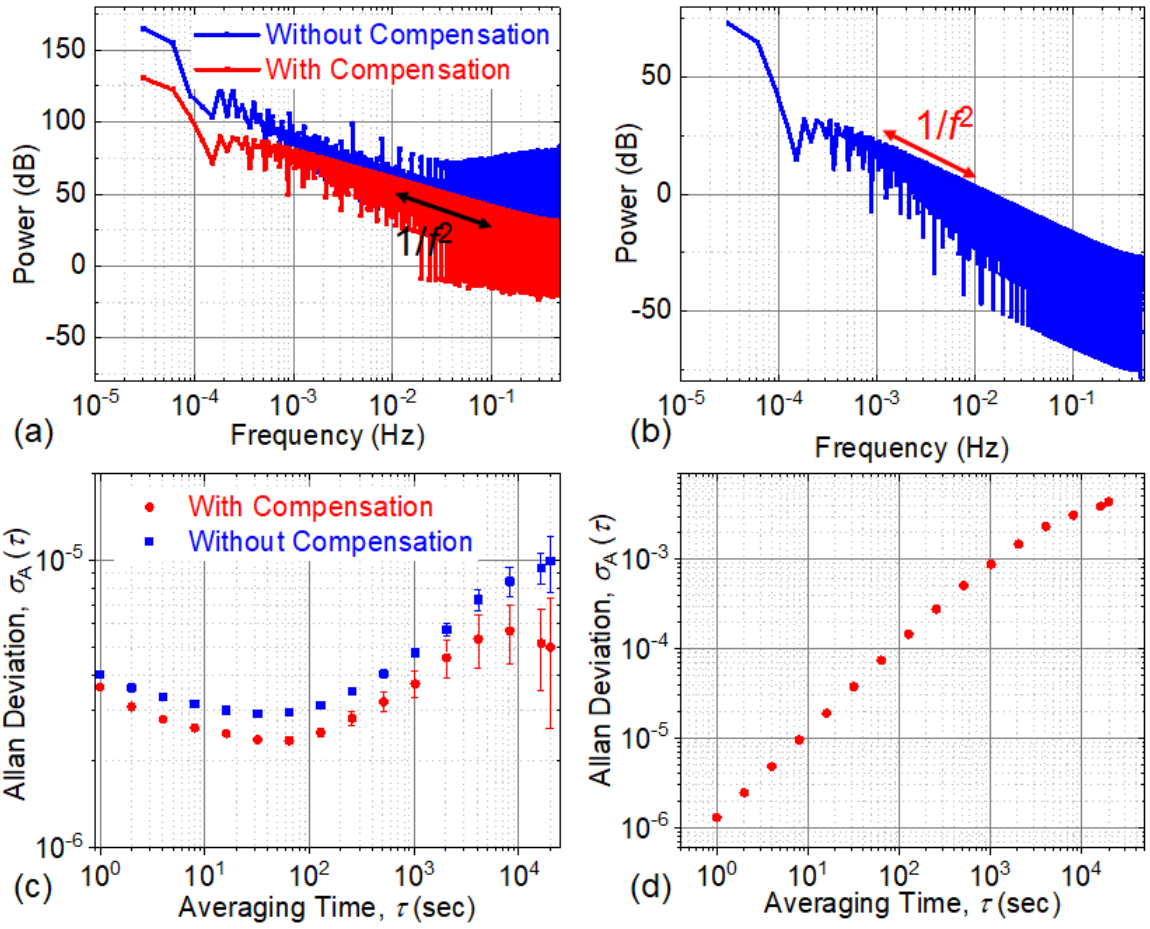

Figure S2. Power spectral density (PSD) and stability estimation: (**a**) PSD of uncompensated and compensated oscillation frequency; (**b**) PSD of ambient temperature fluctuations; (**c**) Allan deviation of uncompensated and compensated oscillation frequency; and (**d**) Allan deviation of the temperature data shown in (**b**).

S3. Effect of Varying Temperature on Uncompensated & Temperature Compensated Oscillators

Figure S3 shows the measured traces of oscillator frequency as a function of time, as the device temperature is varied, for both the oscillators with and without temperature compensation. At each set temperature value, the temperature induced fractional frequency shift (in ppm) and its time-evolution trace, is extracted using the following formula

$$\text{Fractional Frequency Shift}\left(t,T_{Stage}\right)=\frac{f\left(t,T_{Stage}\right)-f_0}{f_0}\times 10^6, \quad (1)$$

where t is time, f_0 is the oscillation frequency at 23.74°C (reference), T_{Stage} is temperature of the device stage controlled by the Peltier module for cooling or the ceramic space heaters for heating. Table S1 displays the mean fractional frequency shift and its standard deviation obtained from the results shown in Figure S3. The results in Table S1 are plotted in Figure 10 in the Main Text.

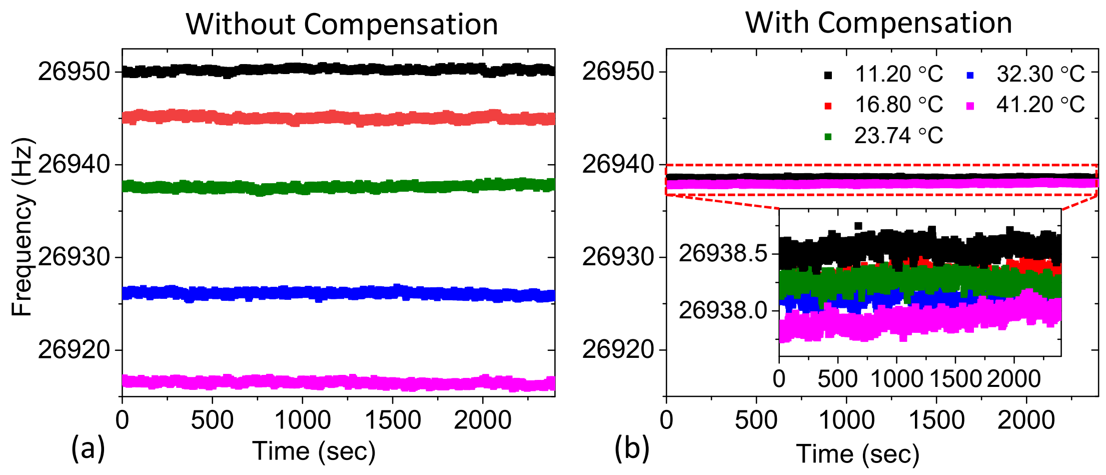

Figure S3. Oscillation frequency traces versus time, measured at five different temperature values set and controlled by the sample heating and cooling modules, for oscillators (**a**) without and (**b**) with temperature compensation, respectively. Inset plot in (**b**) shows zoomed-in view of the red box in (**b**).

Table S1. Fractional frequency shift with and without temperature compensation.

Temperature (°C)	Uncompensated		Compensated	
	Mean Fractional Frequency Shift (ppm)	Standard Deviation (ppm)	Mean Fractional Frequency Shift (ppm)	Standard Deviation (ppm)
11.20	459	6.56	11	2.49
16.80	258	7.87	4	2.04
23.74	0	7.58	0	2.03
32.30	-440	7.63	-10	2.08
41.20	-798	7.62	-14	2.57
